# The memory binding test can anticipate Alzheimer’s disease diagnosis at an early preclinical stage: a longitudinal study in the INSIGHTpreAD cohort

**DOI:** 10.3389/fnagi.2024.1414419

**Published:** 2024-08-08

**Authors:** Filipa Raposo Pereira, Nathalie George, Gianfranco Dalla Barba, Bruno Dubois, Valentina La Corte

**Affiliations:** ^1^Sorbonne Université, Institut du Cerveau - Paris Brain Institute - ICM, INSERM, U 1127, CNRS, UMR 7225, APHP, Hôpital de la Pitié-Salpêtrière, Paris, France; ^2^Department of Neurology, Institute of Memory and Alzheimer’s Disease (IM2A), Pitié-Salpêtrière University Hospital, AP-HP, Paris, France; ^3^Dipartimento di Scienze della Vita, Università degli Studi di Trieste, Trieste, Italy; ^4^Centre of Excellence of Neurodegenerative Disease (CoEN), ICM, CIC Neurosciences, APHP, Department of Neurology, Hôpital Pitié-Salpêtrière, Sorbonne Université, Paris, France; ^5^Laboratoire Mémoire Cerveau et Cognition (UR 7536), Institut de Psychologie, Université Paris Cité, Boulogne Billancourt, France; ^6^Institut Universitaire de France, Paris, France

**Keywords:** Alzheimer’s disease, episodic memory, amyloid-β, free and cued selective reminding test, memory binding test, preclinical Alzheimer’s diagnostic, at-risk, neurodegeneration

## Abstract

**Introduction:**

Anticipating the diagnosis of Alzheimer’s disease (AD) at an early asymptomatic at-risk stage, where therapeutics can more effectively delay conscious cognitive decline, is currently among the biggest challenges in the field. Herein, we aimed to compare the capacity of the Memory Binding Test (MBT) with the official diagnostic tool, the Free and Cued Selective Reminding Test (FCSRT), to anticipate AD diagnosis at an early preclinical stage based on the associative memory component of MBT (binding), suggested as more sensitive to the emergence of subtle episodic memory (EM) deficits (AD hallmark).

**Methods:**

We assessed the tests performance longitudinally (over 5 years) in 263 cognitively-normal elderly individuals at risk of AD (>6 months of subjective memory complaints) using linear mixed-effect models controlled for age, sex, and education. We stratified participants in 2 models: amyloid-β (Aβ)/neurodegeneration (N) model, assessing Aβ burden and neurodegeneration effect [3 groups: controls (Aβ-/N-); stable/N- (Aβ+); stable/N+ (Aβ+)]; and the stable/progressors model, assessing progression to prodromal-AD effect [2 groups: stable (Aβ+); progressors (Aβ+)], based on 15 subjects who progressed to AD during follow-up (excluded once diagnosed).

**Results:**

Aβ burden was associated with significantly less MBT-intrusions, while Aβ burden and neurodegeneration together, with the most. Progression status had a strong negative effect on both tests performance. When compared with the FCSRT, the MBT seems to anticipate diagnosis based on a worst performance in a higher number of scores (including binding) in at least a year.

**Discussion:**

Anticipation of diagnosis to an asymptomatic at-risk stage, while participants remain cognitively-normal according to FCSRT cut-offs and unaware of objective EM deficits, has the potential to delay the onset of AD-linked cognitive decline by applying promising therapeutics before decline becomes too advanced.

## 1 Introduction

Alzheimer’s disease (AD) is the most prevalent form of dementia worldwide and represents a major public health problem in aging populations (Long et al., 2023). Typical AD is primarily characterized as a ‘memory condition’ where deficits in episodic memory (EM), the first cognitive symptom to appear and the more predictive of incident dementia, begin being self-perceived as subjective memory complaints (SMC) by some individuals, in several years preceding diagnosis (Sarazin et al., 2007; Grober et al., 2010; Dubois et al., 2016, 2018; Papp et al., 2017). However, EM is a continuum of memory processes that starts with encoding a new episode by matching an object, location, or event, with a context (i.e., any spatial, temporal environmental, or cognitive feature;Grober et al., 2000; Dubois and Albert, 2004; Sarazin et al., 2007, 2010; Gramunt et al., 2015; Cerami et al., 2017). The episode is then stored for retrieval at any given time (Grober et al., 2000; Dubois and Albert, 2004; Sarazin et al., 2007, 2010; Gramunt et al., 2015; Cerami et al., 2017). In AD, the first deficits that appear tend to be in encoding and retrieval processes linked to executive dysfunction, while storage deficits that underlie the AD clinical phenotype (i.e., amnesic hippocampal syndrome/AHS), are detected only at later preclinical stages (Sarazin et al., 2007, 2010; Grober et al., 2010; Gramunt et al., 2015; Papp et al., 2015, 2017; Dubois et al., 2016; Cerami et al., 2017). However, an essential feature of EM, ‘binding,’ that can be defined as a measure of associative memory consisting in encoding and remembering as a coherent whole the independent different features of an episode, is often overlooked and it remains largely understudied and under-evaluated in clinical assessment (Rentz et al., 2010; Gramunt et al., 2015; Papp et al., 2015; Buschke et al., 2017). Existing evidence suggests that binding, which is also rooted in the hippocampus, is more sensitive to subtle EM alterations when these begin appearing at earlier preclinical stages (Rentz et al., 2010; Gramunt et al., 2015; Papp et al., 2015; Buschke et al., 2017). Research on AD has further demonstrated that patients have a diminished capacity to benefit from item associations, particularly when these are of semantic nature possibly because the deterioration of semantic networks parallels the deterioration of EM networks (Salmon, 2012; Spaan, 2016). Despite this evidence, binding measures are currently absent from the recommended AD-diagnosis composites (Gramunt et al., 2015; Buschke et al., 2017; Papp et al., 2017).

Word list tasks such as the free and cued selective reminder test (FCSRT), are classically used to assess EM in AD (Grober et al., 1987; Auriacombe et al., 2010; Dubois et al., 2014, 2018; Gramunt et al., 2015; Papp et al., 2015; Buschke et al., 2017; Cerami et al., 2017; Loewenstein et al., 2018). These tasks employ cue learning, by pairing a word to be remembered (grapes) with a semantic cue (fruit) that is used at both the encoding and recall stages, to maximize retrieval and minimize individual learning strategies (Grober et al., 1987, 2000; Auriacombe et al., 2010; Papp et al., 2015). Using the FCSRT, AD-linked EM decline is classically diagnosed based on two main scores: (1) the free total recall (FR) score, measuring the accessibility of information and the first to deteriorate, 6–7 years prior to diagnosis; and (2) the total recall (TR; sum of FR and cued recall [CR]) score, measuring availability of information, characteristic of the AD phenotype AHS and deteriorating later, when cues stop overcoming retrieval deficits (Auriacombe et al., 2010; Grober et al., 2010, 2018; Sarazin et al., 2010; Dubois et al., 2014, 2018; Papp et al., 2017; Loewenstein et al., 2018). However, two FCSRT features currently prevent the detection of AD-specific EM deficits in earlier preclinical stages (Auriacombe et al., 2010; Grober et al., 2010; Buschke et al., 2017; Papp et al., 2017; Loewenstein et al., 2018). First, FR deficits in isolation are not AD-specific; only late-appearing TR deficits are neural correlates of AD-AHS (Auriacombe et al., 2010; Grober et al., 2010, 2018; Papp et al., 2017; Loewenstein et al., 2018). Second, as we and groups such as of Gramunt et al. have previously shown, the use of 1 single list of words prompts TR to ceiling levels based on its exclusive comparison with cognitively aging equivalents, by not detecting variation at maximum recall (Papp et al., 2015; Gramunt et al., 2016; Grober et al., 2018; Mowrey et al., 2018). As such, the current challenge remains in anticipating diagnosis to an early preclinical stage, when individuals are asymptomatic at risk and before EM decline is already on the ongoing path to clinical symptomatology (i.e., later preclinical stages; Gramunt et al., 2015, 2016; Papp et al., 2017).

The Memory Binding Test (MBT) is an alternative tool to address this challenge. This test uses two lists of words to assess semantic binding by combining one item from each list with a common cue (item1-cue-item2; Buschke, 2014; Gramunt et al., 2015, 2016; Papp et al., 2015; Buschke et al., 2017). As in the FCSRT, the MBT provides FR and TR equivalent scores but adds a binding component, underlying the capacity to remember, as a coherent whole, the different aspects of an episode (i.e., associative memory; paired condition; Auriacombe et al., 2010; Rentz et al., 2010; Gramunt et al., 2015, 2016; Papp et al., 2015; Spaan, 2016; Buschke et al., 2017; Mowrey et al., 2018; Raposo Pereira et al., 2024). As previously mentioned, binding has been suggested to be more sensitive to the subtle emergence of EM alterations at the early preclinical stage, whereas memory remains normal according to the FCSRT (Rentz et al., 2010; Papp et al., 2015; Gramunt et al., 2016; Buschke et al., 2017; Loewenstein et al., 2018; Mowrey et al., 2018; Gagliardi et al., 2019; Cecchini et al., 2021; Raposo Pereira et al., 2024). Moreover, evidence has shown that the MBT can predict incident dementia and distinguish cognitive aging from amnesic mild cognitive impairment, and from AD, at an earlier preclinical stage (Papp et al., 2015; Gramunt et al., 2016; Mowrey et al., 2016, 2018; Buschke et al., 2017; Raposo Pereira et al., 2024). This is largely centered on the use of two lists of words that eliminate TR ceiling levels by permitting variations at maximum recall and allowing comparison with the individual’s performance (controls or AD) and their initial capacity, which in turn anticipates the detection of TR deficits (AD phenotype) in the paired condition (Dubois et al., 2014; Gramunt et al., 2016; Buschke et al., 2017; Loewenstein et al., 2018). As such, semantic binding has emerged as a potential behavioral marker of AD.

Nevertheless, anticipating an AD diagnosis to the asymptomatic at-risk stage is challenging. For instance, SMC are the first conscious report of EM alterations in AD but are also common in cognitive aging (Auriacombe et al., 2010; Gramunt et al., 2015; Loewenstein et al., 2018). Therefore, identifying their origin, while levels of EM remain within the official cut-offs of the FCSRT and normal according to the perception of the subject and their companion, is crucial (Auriacombe et al., 2010; Sperling et al., 2011; Gramunt et al., 2015, 2016; Dubois et al., 2016; Loewenstein et al., 2018). Furthermore, EM deficits have been associated with AD position emission tomography (PET) biomarkers, which are harbored in the brain years before the diagnosis or the manifestation of cognitive symptoms (Rentz et al., 2010; Sperling et al., 2011; Papp et al., 2015, 2017; Bilgel et al., 2018; Dubois et al., 2018; Mormino and Papp, 2018; Chipi et al., 2019). Such is the case of amyloid-β (Aβ) burden (i.e., deposition of Aβ plaques), a diagnostic criterion and tendentially the first biological process to degenerate, and neurodegeneration (i.e., hypometabolism), appearing after Aβ burden on the AD continuum (Rentz et al., 2010; Sperling et al., 2011; Papp et al., 2015, 2017; Bilgel et al., 2018; Dubois et al., 2018; Mormino and Papp, 2018; Chipi et al., 2019). Moreover, when present simultaneously with conscious SMC, these AD-biomarkers correlate with an increase in the amount and severity of EM deficits (Sperling et al., 2011; Papp et al., 2015, 2017; Bilgel et al., 2018; Mormino and Papp, 2018; Chipi et al., 2019). However, not all patients who present with biomarkers and SMC develop AD (Auriacombe et al., 2010; Bilgel et al., 2018). Nevertheless, evidence suggests that the asymptomatic at-risk stage of AD is a time window with a higher chance of successfully receiving therapeutics that can delay cognitive decline (Auriacombe et al., 2010; Gramunt et al., 2015; Papp et al., 2017; Bilgel et al., 2018).

While the FCSRT can detect and characterize typical EM deficits in AD at later preclinical stages, mainly based on one score (TR), the MBT is capable of detecting subtle EM deficits when they start appearing at asymptomatic at-risk stages (early preclinical), based on at least two types of binding scores (Auriacombe et al., 2010; Gramunt et al., 2016; Mowrey et al., 2016; Buschke et al., 2017; Papp et al., 2017; Grober et al., 2018; Raposo Pereira et al., 2024). Moreover, although the normative and psychometric properties of the FCSRT have been well-validated, the validity of the MBT remains to be fully confirmed (Auriacombe et al., 2010; Gramunt et al., 2015; Papp et al., 2017; Loewenstein et al., 2018; Mowrey et al., 2018).

In a previous exploratory study, we have retrospectively assessed the diagnostic capacity of the MBT over a 5-year follow-up period in a selected sample of 45 asymptomatic elderly participants at-risk for AD (>6 months of SMC) from the INSIGHTpreAD cohort, who were carefully matched in terms of age, sex, education level, and level of Aβ burden and neurodegeneration (Raposo Pereira et al., 2024). In this study results showed that in relation to the FCSRT, the MBT could anticipate the detection of EM deficits linked to the progression to AD, but Aβ burden did not influence MBT performance (Raposo Pereira et al., 2024). Building on this work here we assessed if the diagnostic capacity of the MBT is maintain in a broader sample of 263 participants from the same cohort, with different levels of AD-biomarkers, with the aim of: (1) assessing the effect of Aβ burden and neurodegeneration on the MBT performance; (2) assessing the effect of progression to prodromal-AD on MBT performance; (3) comparing the performances of MBT and FCSRT in their main parallel scores (FR and TR/total binding), comparing the initial session at which significant decline in EM starts occurring, whether binding decline can be detected earlier, and their diagnosis accuracy (Raposo Pereira et al., 2024).

## 2 Methods

### 2.1 Participants

In this study, we initially included 318 cognitively normal elderly individuals at risk of AD from the multimodal INSIGHTpreAD cohort (Dubois et al., 2018; Raposo Pereira et al., 2024). These participants were followed up for 5 years and matched for age, education level, and sex during recruitment. ‘At-risk,’ was defined here as ≥6 months of self-reported SMC in consultation, with or without the presence of Aβ burden (SUVr>0.7918, measured with Aβ PET) and neurodegeneration (SUVr < 2.27, measured with ^18^F-fluorodeoxyglucose [^18^F-FDG] PET; Dubois et al., 2018; Habert et al., 2017; Jack et al., 2017; Raposo Pereira et al., 2024). Participants were recruited monocentrically from the Institute for Memory and Alzheimer’s disease (IM2A) at the Pitié-Salpêtrière Hospital, Paris. The inclusion criteria were: age 70–85 years, unimpaired cognition (i.e., mini-mental state examination [MMSE], total ≥ 27/30; FCSRT, TR ≥ 41/48); clinical dementia rating = 0; normal visual and auditory capacity (Folstein et al., 1975; Grober et al., 1987; Morris, 1993). The exclusion criteria were as follows: currently under guardianship or in a nursing facility; diagnosis of AD or other neurological diseases, illiteracy, and inability to undergo neural MRI. Persistent cognitive decline in two consecutive and relevant neuropsychological assessments (i.e., MMSE, CDR, and/or FCSRT-TR) in individuals presenting with Aβ burden (i.e., AD biomarker) and AHS (i.e., AD phenotype) suggested the onset of AD-symptomatology, and warned of a detailed diagnostic assessment. During follow-up, 15 subjects were diagnosed with prodromal AD by two neurologists, a neuropsychologist, and a neuroimaging expert, indicating their automatic removal from the INSIGHTpreAD cohort (Dubois et al., 2018). The same outcome was applied in cases diagnosed with any other relevant condition. All participants agreed to participate freely by signing consent and indicated their willingness to commit to the longevity of the study after the study conditions were presented. However, dropouts were possible at all times.

### 2.2 Ethics

All aspects of this study were designed in full compliance with French law n° 2004–806 (9th of August 2004), Good Clinical Practice principles (I.C.H version 4 of May 1, 1996, and the decision of 24th November 2006), and the guidelines of the Helsinki Declaration (Ethical Principles for Medical Research involving Human Subjects, Tokyo 2004). The Ethics Committee of Pitie-Salpêtrière University Hospital approved the INSIGHT-PreAD protocol and the INSIGHT-preAD scientific committee approved this project (Dubois et al., 2018).

### 2.3 Procedure

The observational INSIGHT-preAD longitudinal cohort (5-years), included a vast number of disciplines aimed at broadly characterizing the at-risk phase (SMC >6 months) of cognitively normal elderly individuals and isolating markers of progression to prodromal AD (Dubois et al., 2018). Data collection included one full experimental day with breaks between assessments. In this study only a subset of data, comprising PET imaging scans to assess Aβ burden and neurodegeneration (collected at baseline, in the afternoon), and neuropsychological evaluation (collected at baseline and at every 12 months, in the morning) were considered. The participants were assessed clinically every 6 months.

### 2.4 PET acquisition

The regional standard uptake value ratio (SUVr) of either Aβ burden or neurodegeneration was collected with the Philips Gemini GXL CR-PET scanner.

Aβ burden SUVr was measured 50 min following the injection of 370 MBq (10 mCi) ^18^F-florbetapir (marker of neocortical deposition of Aβ plaques; Habert et al., 2017). Acquisition was based on the Jaszczack’s and 3D-Hoffman’s phantoms measurements with the following parameters: frames = 3×5; acquisition matrix = 128×128; voxel size = 2x2x2 mm3. The LOR-RAMLA algorithm with 10 iterations was used in image reconstruction, a lambda relaxation parameter of 0.7 was used to reduce noise, with all the adjustments being introduced in the reconstruction (Habert et al., 2017). The following regions of interest (ROIs) were used to collect regional values: the left and right precuneus, posterior cingulum, anterior cingulum, associative parietal cortex, associative temporal cortex, orbitofrontal cortex, and whole cerebellum plus pons (reference region; Habert et al., 2017). An SUVr > 0.7918 cut-off was calculated for the INSIGHTpreAD through a linear conversion of the CAEN method (i.e., mean ROIs SUVr averaged to the reference region) to distinguish the Aβ positive status (Aβ+; Habert et al., 2017).

The acquisition parameters used of the FDG PET were similar to the ones described above used on the amyloid PET (Habert et al., 2017; Jack et al., 2017; Dubois et al., 2018). The FDG SUVr was measured 30 min following the injection of 2 MBq/kg ^18^F-FDG (a marker of cortical glucose uptake; Habert et al., 2017; Jack et al., 2017; Dubois et al., 2018). Regional values were collected from the following ROIs: posterior cingulate cortex, inferior parietal lobe, precuneus, inferior temporal gyrus, and pons (reference region; Habert et al., 2017; Jack et al., 2017). A similar linear method as described above led to a cut-off of SUVr < 2.27, defining the neurodegeneration-positive (N+) status as an expression of deficient uptake of cortical glucose, that is, hypometabolism (Habert et al., 2017; Jack et al., 2017).

### 2.5 Neuropsychological assessments

French sociocultural (NSC) level was used as a proxy for education, scored from 1 (illiterate) to 8 (at least 2 years of higher education after a bachelor’s degree). Cognitive impairment and/or dementia were suggested with an MMSE score < 27/30, requiring a more detailed cognitive examination (Folstein et al., 1975; Dubois et al., 2018). Executive impairment was suggested with a Frontal Assessment Battery (FAB) score of ≤12/18, characteristic of AD and distinctive of AD from other frontal dementias (Dubois et al., 2000, 2018). Abnormal SMC were considered for a score ≥ 15 in the 15-item French version of the McNair Frequency of Forgetting Questionnaire (McNair Questionnaire), to assess the conscious severity and frequency of complaints in the participants (from M0) and their companions (from session M12; since this was their first SMC recording session; McNair and Kahn, 1983; Van Der Linden et al., 2004; Dubois et al., 2018).

### 2.6 The free and cued selective reminding test

The FCSRT begins with the presentation of four consecutive cards with four words each (amounting to one list of 16 words in total; Grober et al., 1987). During the learning and encoding phases, participants were required to orally recognize the word that corresponded to the respective semantic cue (Grober et al., 1987). During the recall phase, participants had to perform the immediate recall (IR) of a maximum number of words (three trials), intercalating with CR trials, where semantic cues were presented for the words not remembered during IR until the retrieval of all missing items (Grober et al., 1987). FR (sum of the three IR trials; 0–48), and TR (sum of each IR plus CR trials; 0–48), are the official FCSRT scores for AD diagnosis (Grober et al., 1987). Each FR-TR pair of trials was intercalated with a 20 s backward counting interference task (Grober et al., 1987). A TR ≤ 41 suggests temporal-limbic amnesia (i.e., AHS). Delayed FR (DFR) and delayed TR (DTR) trials based on the same semantic content were assessed 20 min later (Grober et al., 1987).

### 2.7 Memory binding test

As in the FCSRT, the MBT (former memory capacity test) requires the learning of 2 lists of 16 words, which introduces binding using 16 unifying semantic cues (e.g., semantic cue = insect, word ListA = flea, word ListB = ant; Grober et al., 1987, 2000; Buschke, 2014). The MBT starts with the encoding of ListA, CR of ListA items by orally presenting the corresponding cues (CRa; 0–16 score; 5 s), encoding of ListB, and similar CR of ListB items (CRb; Buschke, 2014). The paired condition (ListA+B) was followed by the presentation of cues to obtain the TR of both lists (TCR_A + B_; Buschke, 2014). The origin (ListA or B) of each word from the wordA-wordB pair was subsequently required to orally originate the source memory recall (SMR; %; Buschke, 2014). This was followed by the recall of items from both lists in any order (10 s each; Buschke, 2014). Subsequently, the total number of items correctly recalled in association with each cue (TIP; 0–32 score) and the number of corrected pairs of CR (PIP; 0–16 score) were obtained (Buschke, 2014). This was followed by the maximum FR of the items (from both lists) in any order (0–32 score; Buschke, 2014). DFR (0–32 score), delayed TIP (DTIP; 0–32 score), and delayed SMR (DSMR; %) were assessed after 30 min (Buschke, 2014). The cues were presented similarly (Buschke, 2014). Intrusions were recorded in different modalities: total (T_intr); semantically related (extra-list_intr); semantically unrelated (extra-category_intr); and words originating from List A are presented when List B is being recalled (prior-list_intr; Buschke, 2014).

### 2.8 Statistical analysis

Neuropsychological data collected every 12 months over 5 years from baseline (M0, M12, M24, M36, M48, and M60) were assessed statistically using R studio (4.2.1 software version; https://www.R-project.org/).

#### 2.8.1 Demographic and clinical characterization of the groups at baseline (M0)

At baseline we assessed age, sex, education level, SMC reported by participants, and SMC reported by their companions (session M12) separately, to evaluate potential differences between the 5 groups of interest in these variables. For this we used the 5-groups as a between-subject factor in one-way Welch analyses of variance (ANOVA) for its capacity to account for unequal variance of different group sizes (car R package, version 3.1.0). Similarly, we tested differences in Aβ-SUVr between the Aβ + groups, and differences in FDG-SUVr between the N+ groups. We further assessed neuropsychological tests included as an inclusion criterion at baseline (i.e., MMSE, FCSRT_TR, FAB) with general linear models (Glm; ‘stats’ R package, version 4.2.1), using groups-of-interest as between-subject fixed-factor, and age, sex, and education level as fixed-effect covariates to accommodate for their confounding effect on cognitive performance. Age was considered a numerical variable (mean centered at baseline), while sex and education level were considered categorical variables. We assessed *post hoc* comparisons using Games-Howell tests when main effects were observed (rstatix R package, version 0.7.0), corrected for multiple comparisons, and significance was considered at *p* < 0.05.

#### 2.8.2 FCSRT and MBT analysis

To investigate our research questions, we created two statistical models based on different between-subject factors. One model aimed at assessing the effect of Aβ burden and neurodegeneration (the Aβ/N model), considering 3 groups of non-progressors to prodromal AD. One group of controls (presence of SMC but without AD-biomarkers), and two groups of stable participants (presence of SMC and at least one AD-biomarker) as follows: stable/N- (Aβ+/N-), and stable/N+ (Aβ+/N+). We tested the effect of Aβ burden by contrasting the controls vs. stable/N- groups and the effect of neurodegeneration by contrasting the stable/N- vs. stable/N+ groups as fixed-effects. The other model aimed at assessing the effect of progression to prodromal AD based on the 15 participants that were diagnosed during the follow-up period but considered only until diagnosis (the stable/progressors model), considering only Aβ + participants (criteria in AD-diagnosis) classified in two groups: stable (N- and N+) and progressor (N- and N+) groups. We further tested progression to prodromal AD as a fixed-effect (stable vs. progressor group contrast). Linear mixed-effects (lmer) models were used to longitudinally assess outcome scores, accounting for missing data (lme4 R package, version 1.1.29). ‘Session’ was introduced as a repeated-measures fixed-effect (6 levels: M0, M12, M24, M36, M48, M60), as well as group status (3 groups = Aβ/N model, 2 groups = stable/progressors model), and the interaction between them was considered (session*group_status). Participants were introduced as a random-effect with a random intercept per subject (‘1| participant iD’). Age, sex, and educational level were further introduced as covariates of no interest to minimize potential confounding effects. Pairwise post-hocs were performed for our effects of interest (i.e., Aβ burden, neurodegeneration, or progression to prodromal AD) with emmeans (R package, version 1.7.5) and corrected for multiple comparisons when main effects were significant. Statistical significance was set at *p* < 0.05.

#### 2.8.3 Receiver operating characteristic curve analysis

We calculated ROC curves at baseline (session M0; pROC package v1.18.5; R 4.2.1 software version; https://www.R-project.org) to compare the diagnostic capacity of the FCSRT versus MBT in discriminating among at-risk level participants, cognitively normal but with at least 1 biomarker, who will progress to AD within +/− 5-years. To test diagnose accuracy, we calculated the area under the ROC curve (AUC) for each main score of each test, comparing 2×2 the score of one test with its equivalent in the other test, and we used the DeLong’s test to compare the AUC of each score based on their 95% confidence intervals. The Youden index (J) method was used to outline the optimal cut-off value of each score based on the best tradeoff between sensitivity (true-positive rate; correct diagnose of AD) and specificity (false-positive rate or noise; correct clearing of AD). However, we are aware that the ‘optimal cut-off scores’ can change according with the characteristics of the sample and the tradeoff between sensitivity/specificity.

## 3 Results

### 3.1 Demographic and clinical characterization of the groups at baseline (M0)

**Figure 1:**
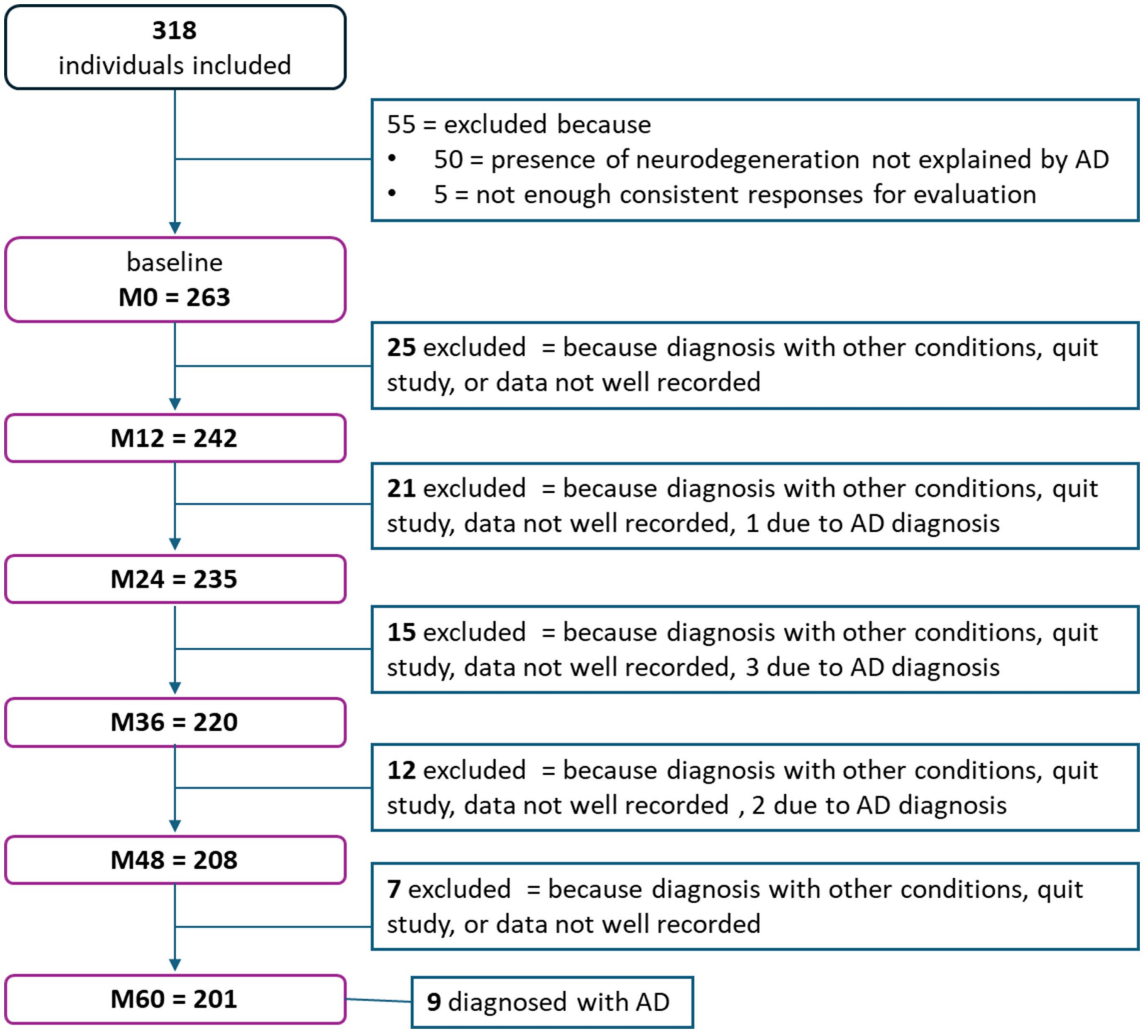
Flowchart representing the number of participants that either left the study or were excluded per session.

The INSIGHTpreAD cohort included 318 cognitively normal elderly individuals at risk of AD (>6 months of SMC). Of these, 50 participants with neurodegeneration and without Aβ burden were excluded as we could not link their source of neurodegeneration to AD. All participants were longitudinally assessed retrospectively; however, owing to different causes, some were dismissed during follow-up (i.e., recording problems, quitting the study, or death). This resulted in the following number of subjects per session: 263 at M0, 242 at M12, 235 at M24, 220 at M36, 208 at M48, and 201 at M60 (Figure 1). Fifteen participants progressed to a confirmed AD diagnosis during follow-up and were removed from the study (diagnoses per session: M18 = 1, M24 = 3, M36 = 1, M42 = 1, M60 = 9). Hence, at baseline the groups comprised the following number of participants: 175 controls; 57 stable/N-; 16 stable/N+; 15 progressors. For the stable/progressors model, we included 73 stable and 15 progressor participants. On average, at baseline participants were 76 ± 3.5 years old (mean ± standard deviation [SD]), were predominantly women (*n* = 173; 65.5%), and were highly educated (mean = 6.21 ± 2.0; i.e., bachelor undergraduate level). Only sex differed significantly between the groups (*p* = 0.018), since there was a predominance of females, normal in this type of population. Age and education did not differ significantly between the groups (Table 1). The AβSUVr was significantly different between the Aβ + groups [*F*(3, 14.8) = 6.2, *p* = 0.006] as a result of higher AβSUVr on the progressors vs. the stable group (*p* = 0.0009; post-hoc analysis). All groups met the psychometric thresholds for the required inclusion scores: 28.7 ± 1.0 MMSE_total score (≥27/30), 16.4 ± 1.7 FAB_total score (≥12/18), 46.1 ± 1.9 FCSRT_TR (≥41/48), 13.0 ± 6.2 McNair total_participant (abnormal = SMC ≥ 15), 8.1 ± 6.2 McNair total_companions (abnormal = SMC ≥ 15). Significant differences were further observed at baseline in the FCSRT_FR [*X*^2^(4) = 12.7, *p* = 0.013], driven by a significantly lower FR in progressors than in stable participants (*p* = 0.003).

**Table 1:** Demographic and clinical characterization of the groups of interest at baseline.

	Controls	Stable		Progressors			
	Aβ-/N- (*n* = 175)	Aβ+/N- (*n* = 57)	Aβ+/N+ (*n* = 16)	Aβ+/N- (*n* = 8)	Aβ+/N+ (*n* = 7)		
	Mean ± SD	Mean ± SD	Mean ± SD	Mean ± SD	Mean ± SD	*F*_*df1*, df2_ = value/*X^2^*_df1_ *=* value	*p*
Age	75.5 ± 3.4	76.8 ± 3.3	76.4 ± 3.9	77.8 ± 2.4	77.0 ± 4.7	F_4, 22.2_ = 2.6	0.07^a^
Education Level	6.3 ± 2.0	5.7 ± 2.0	6.9 ± 1.7	6.3 ± 2.2	6.6 ± 2.4	F_4, 22.0_ = 1.7	0.18^a^
Sex (%)	*F* = 117 (67); M = 58 (33)	*F* = 42 (73); M = 15 (26)	F = 6 (38); M = 10 (63)	*F* = 6 (75); M = 2 (25)	*F* = 2 (29); M = 5 (71)	*X^2^*_4_ = 11.9	0.018^**,**a**^
Aβ SUVr	0.7 ± 0.1	1.0 ± 0.1	1.1 ± 0.2	1.2 ± 0.2	1.2 ± 0.2	*F*_*3*, 14.8_ = 6.2	0.006^***,**a**,**c1**,**1**^
Neurodegeneration SUVr	2.6 ± 0.2	2.5 ± 0.2	2.1 ± 0.1	2.5 ± 0.2	2.1 ± 0.1	*F*_1, 15.1_ = 0.0009	0.98^a,2^
MMSE_total (≥27/30) [min-max]	28.7 ± 1.0 [27–30]	28.5 ± 0.9 [27–30]	28.6 ± 1.0 [27–30]	28.1 ± 0.8 [27–29]	28.3 ± 0.5 [28–30]	*X^2^*_4_ = 3.5	0.48^b^
FCSRT_free recall [min-max]	30.4 ± 5.3 [13–16]	30.5 ± 4.8 [17–43]	28.5 ± 4.1 [21–39]	24.4 ± 5.8 [18–34]	24.4 ± 7.3 [14–36]	*X^2^*_4_ = 12.7	0.013^**,**b**,**c1**^
FCSRT_total_recall (≥41/48) [min-max]	46.2 ± 1.9 [41–48]	46.4 ± 1.8 [41–48]	46.1 ± 1.5 [43–48]	44.6 ± 1.9 [41–47]	44.7 ± 2. [41–48]	*X^2^*_4_ = 7.1	0.13^b^
FAB (12/18) [min-max]	16.5 ± 1.7 [10–18]	16.0 ± 1.7 [11–18]	16.3 ± 1.2 [14–18]	16.3 ± 1.5 [14–18]	15.7 ± 2.7 [12–18]	*X^2^*_4_ = 4.0	0.40^b^
M. complaints_participant (normal < 15)	13.4 ± 6.6	12.9 ± 5.3	10.6 ± 4.9	14.0 ± 6.0	9.3 ± 5.2	*F*_*4*, 22.6_ = 1.9	0.15^a^
M. complaints_companions (normal <15)	7.7 ± 6.2	8.4 ± 5.5	9.5 ± 6.8	6.8 ± 4.6	12.7 ± 9.1	*F*_4, 17.8_ = 0.7	0.58^a^

Aβ SUVr = β-Amyloid Standardized Uptake Value ratio; *N* = neurodegeneration Standardized Uptake Value ratio; SD = Standard Deviation; df = degrees of freedom; MMSE = Mini Mental State Examination; FCSRT = Free and Cued Selective Reminding Test; FAB = Frontal Assessment Battery; M = memory *X*^2^ = chi-square. p-values are reported for the main effect of group on each variable between the 5-groups at session M0: *** *p* < 0.001; ** *p* < 0.01; * *p* < 0.05. Statistical analysis was performed as follows: (a) Welch ANOVA (reporting F-values); (b) general linear model (GLM reporting *X*^2^-values); (c) post-hoc Games-Howell tests were used when the main effect of group was significant. Significant differences were found on (c1) progression-to-prodromal-AD effect: Aβ_SUVr: p = 0.0009 [progressors > stable], FCSRT_free recall: p = 0.003 [progressors < stable]. The superscript numbers indicate which groups were compared in selected outcome variables: 1 = only Aβ positive groups 2 = only neurodegeneration positive groups.

### 3.2 Aβ/N model

**Figure 2:**
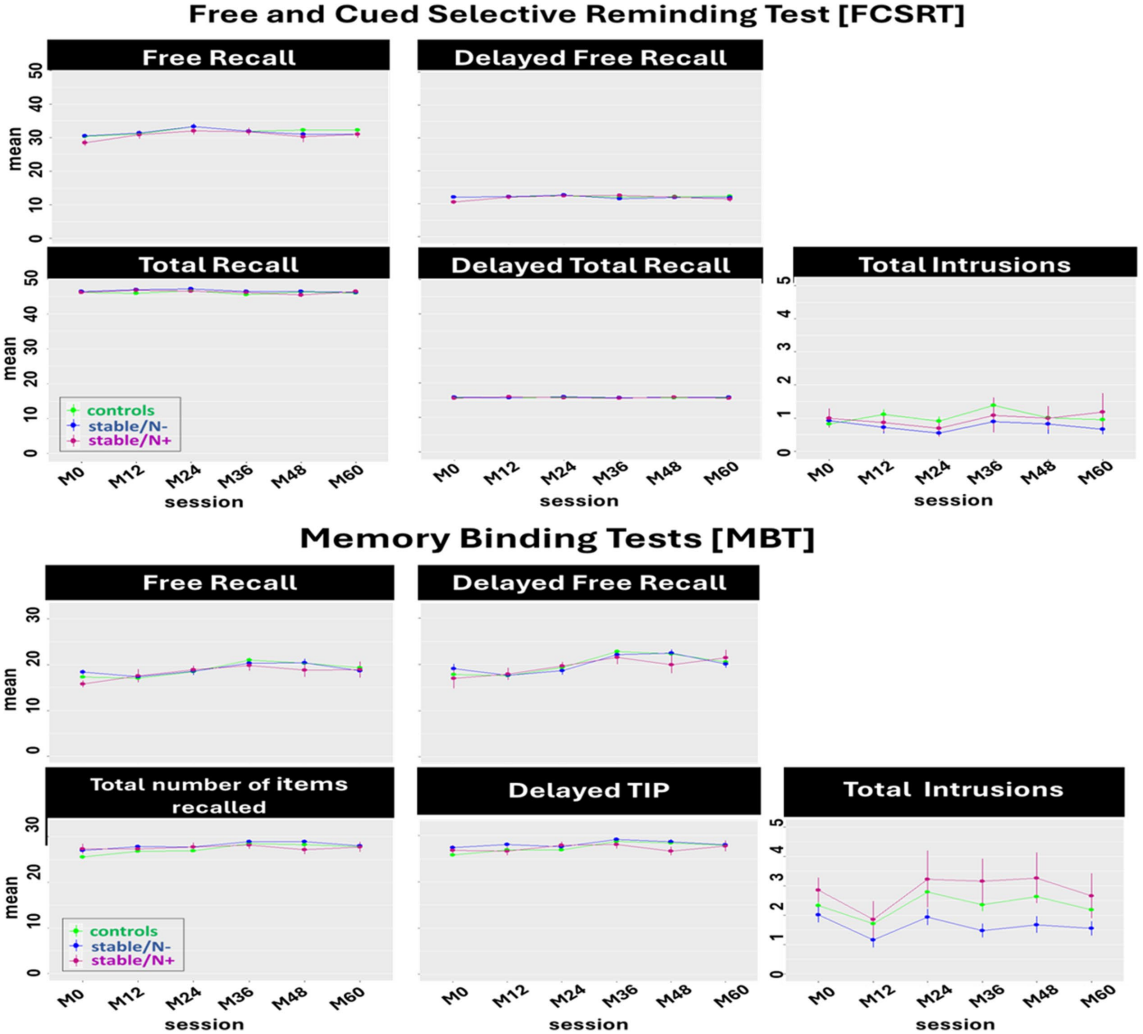
Effect of Aβ burden and neurodegeneration on the longitudinal performance of the Free and Cued Selective Reminding test [FCSRT] and the Memory Binding Test [MBT] main scores: Between-group comparison (controls, stable/N-, stable/N+) of the mean of each main score per session. Only a tendency was observed for a significant interaction on the Delayed TIP (*p* = 0.05) but post-hoc tests showed no significant Aβ burden or neurodegeneration effect.

In this model we included 3 groups (i.e., controls, stable/N-, and stable/N+) to assess the effect of Aβ burden (i.e., controls vs. stable/N-) and neurodegeneration (i.e., stable/N- vs. stable/N+) on the MBT and FCSRT longitudinal performance (Figure 2).

In the FCSRT, we identified a main effect of session on the FR_A_ [*F*(5, 1, 038) = 7.07, *p* < 0.0001], TR_A_ [*F*(5, 1, 057) = 2.63, *p* = 0.02], and DFR_A_ [*F*(5, 1, 047) = 3.29, *p* = 0.006]. Post-hoc tests showed a significant decline in FR_A_ and TR_A_ scores (from session M24-M48, *p* = 0.01; and M24-M36, *p* = 0.05, respectively), and a significant increase in DFR_A_ (session M0-M24, *p* = 0.01). No significant group effects were observed in the performance of the FCSRT.

In the MBT, we found a main effect of session in the FR_A + B_ [*F*(5, 1, 020) = 10.52, *p* < 0.0001], TIP_A + B_ [*F*(5, 885) = 5.49, *p* < 0.0001], DFR_A + B_ [*F*(5, 888) =17.86, *p* < 0.0001], DTIP_A + B_ [*F*(5, 884) = 5.30, *p* < 0.0001], and T_intr_A + B_ [*F*(5, 1, 026) = 3.06, *p* = 0.009]. This translated to a significant increase from session M0-M48 on the FR_A + B_, TIP_A + B_, and DFR_A + B_ (*p* = 0.0001, *p* = 0.003, and *p* = 0.0001, respectively), from sessions M0-M36 on DTIP_A + B_ (*p* = 0.0001), and from sessions M12-48 on T_intr_A + B_ (*p* = 0.03). There was also a main effect of group in T_intr_A + B_ (*F*(2, 237) =5.13, *p* = 0.007), where post-hoc tests showed less intrusions in the stable/N- group than in the controls (*p* = 0.01; Aβ burden effect), and less intrusions in the stable/N- group than in the stable/N+ group (*p* = 0.04; neurodegeneration effect).

Overall, neither Aβ burden nor neurodegeneration status seem to exert any other effect in FCSRT and MBT performance at this at-risk preclinical stage (Table 2).

**Table 2:** Comparison of the 3 groups of the Aβ/N model to assess the effect of Aβ burden and neurodegeneration on the longitudinal performance of the free and cued selective reminding test (FCSRT) and memory binding test (MBT) main scores.

	M_0_			M_60_				
	Controls	Stable/N-	Stable/N+	Controls	Stable/N-	Stable/N+		
Score name	Aβ- (*n* = 175)	Aβ+ (*n* = 57)	Aβ+ (*n* = 16)	Aβ− (*n* = 139)	Aβ+ (*n* = 41)	Aβ+ (*n* = 11)		
	Mean ± SD	Mean ± SD	Mean ± SD	Mean ± SD	Mean ± SD	Mean ± SD	*F*_df1, df2_ *=* value; *p* = value	
**FCSRT**								
FR_A_ [0–48]	30.4 ± 5.3	30.5 ± 4.8	28.5 ± 4.1	32.2 ± 6.5	31.0 ± 5.7	31.0 ± 3.6	**gr:** *F*_2, 240_ = 0.4; *p* = 0.69 **sess:** *F*_5, 1,038_ = 7.1; *p* < 0.001^***^ gr*sess: *F*_10, 1,037_ = 1.3; *p* = 0.22	
DFR_A_ [0–48]	12.1 ± 2.1	12.1 ± 2.1	10.6 ± 2.4	12.3 ± 2.3	12.0 ± 2.3	11.5 ± 3.0	**gr:** *F*_2, 240_ = 0.2; *p* = 0.78 **sess:** *F*_5, 1,047_ = 3.3; *p* = 0.006^**^ gr*sess: *F*_10, 1,045_ = 1.4; *p* = 0.19	
TR_A_ [0–48]	25.5 ± 4.2	46.4 ± 1.8	46.1 ± 1.5	46.0 ± 2.8	46.2 ± 1.9	46.5 ± 1.9	**gr:** *F*_2, 245_ = 2.6; *p* = 0.15 **sess:** *F*_5, 1,057_ = 2.7; *p* = 0.02^**^ gr*sess: *F*_10, 1,054_ = 1.4; *p* = 0.19	
DTR_A_ [0–48]	15.7 ± 0.6	15.8 ± 0.4	15.6 ± 0.8	15.7 ± 0.8	15.8 ± 0.4	11.6 ± 0.7	**gr:** F_2, 242_ = 1.4; *p* = 0.26 **sess:** F_5, 1,063_ = 1.8; *p* = 0.12 gr*sess: F_10, 1,059_ = 0.7; p = 0.69	
T_intr_A_	0.8 ± 1.4	0.9 ± 1.6	1.0 ± 1.2	1.0 ± 1.6	0.7 ± 1.0	1.2 ± 1.9	**gr:** *F*_2, 238_ = 1.0; *p* = 0.35 **sess:** *F*_5, 838_ = 1.0; *p* = 0.40 gr*sess: *F*_10, 836_ = 0.7; *p* = 0.72	
**MBT**								
FR_A + B_ [0–32]	17.3 ± 4.7	18.4 ± 4.1	15.8 ± 2.9	19.3 ± 5.2	18.6 ± 5.1	18.9 ± 5.3	**gr:** *F*_2, 245_ = 0.3; *p* = 0.75 **sess:** *F*_5, 1,020_ = 10.5; *p* < 0.001^***^ gr*sess: *F*_10, 1,017_ = 1.1; *p* = 0.34	
DFR_A + B_ [0–32]	17.8 ± 4.9	19.1 ± 4.8	17.0 ± 5.4	20.5 ± 5.3	20.1 ± 5.1	21.4 ± 5.1	**gr:** *F*_2, 232_ = 0.2; *p* = 0.85 **sess:** *F*_5, 888_ = 17.9; *p* < 0.001^***^ gr*sess: *F*_10, 887_ = 1.2; *p* = 0.27	
TIP_A + B_ [0–32]	25.5 ± 4.2	27.0 ± 2.4	27.3 ± 2.7	27.9 ± 3.4	28.0 ± 3.2	27.8 ± 3.3	**gr:** *F*_2, 229_ = 2.3; *p* = 0.10 **sess:** *F*_5, 885_ = 5.5; *p* < 0.001^***^ gr*sess: *F*_10, 884_ = 1.2; *p* = 0.28	
DTIP_A + B_ [0–32]	25.8 ± 4.2	27.4 ± 2.2	26.7 ± 2.6	27.9 ± 3.5	27.9 ± 3.5	27.8 ± 3.5	**gr:** *F*_2, 228_ = 2.2; *p* = 0.12 **sess:** *F*_5, 884_ = 5.3; *p* < 0.001^***^ gr*sess: F_10, 882_ = 1.8; *p* = 0.05	
T_intr_A + B_	2.3 ± 2.5	2.0 ± 2.0	2.9 ± 1.6	2.1 ± 2.4	1.6 ± 1.5	2.7 ± 2.3	**gr:** *F*_2, 237_ = 5.1; *p* = 0.007^**,a,1^ **sess:** *F*_5, 1,027_ = 3.1; *p* = 0.009^**^ gr*sess: *F*_10, 1,023_ = 0.3; *p* = 0.99	

*N* = neurodegeneration status; Aβ = amyloid-β status; SD = Standard Deviation; df = degrees of freedom; FR = free recall; DFR = delayed free recall; TR = total recall; DTR = delayed total recall; TIP = total number of items recalled in the paired condition (ListA + B); DTIP = delayed TIP; T_intr = Total intrusions; gr = group; sess = session. The values within the brackets represent the range of possible scores per measure. The values within the brackets represent the range of possible scores per measure. The subscript A represents the FCSRT 1 list of words and A + B represents the MBT 2 lists of words. The table represents the longitudinal mean and SD for each group in the Aβ/N model at M0 and M60. F-values, and p-values of the interaction group_status*session corresponding to the longitudinal analysis of the Aβ/N model assessed with linear mixed effect models are represented. Superscript a represents cases where the main effect of group was significant and further assessed with emmeans post hoc analysis (corrected for multiple comparisons). (1) controls > stable/N-: *p* = 0.01; (2) stable/N- < stable/N+: *p* = 0.04.

### 3.3 Stable/progressors model

**Figure 3:**
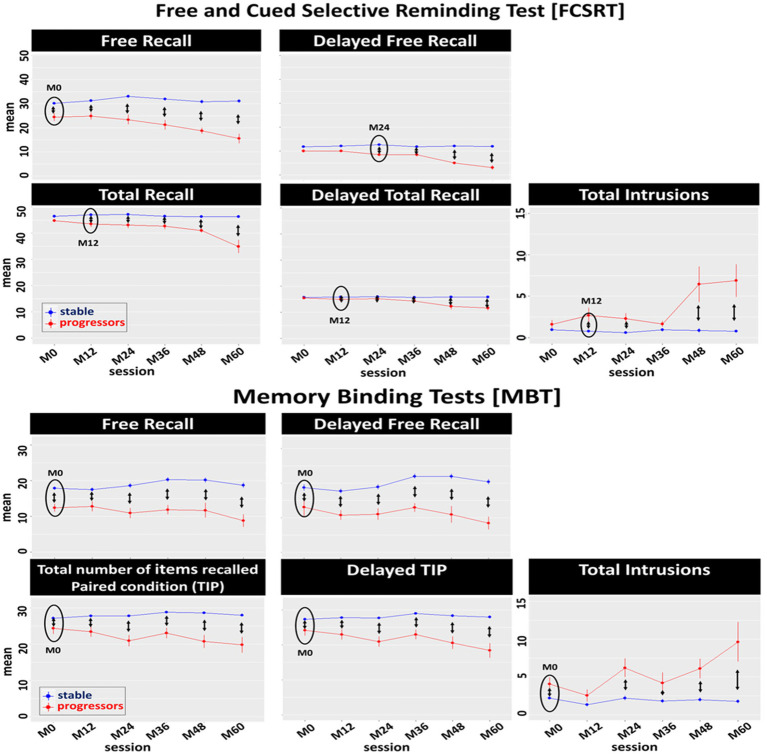
Effect of progression to prodromal AD on the longitudinal performance of the Free and Cued Selective Reminding test (FCSRT) and the Memory Binding Test (MBT) main scores: Between-group comparison (stable, progressors) of the mean of each main score per session. Black circles represent the first session in which significant differences were observed between groups. Double arrows represent significant differences between stable and progressor groups per session.

In this model, we compared the stable and progressor groups to assess the effect of progression to prodromal AD on MBT and FCSRT longitudinal performance (Figure 3).

In the FCSRT, we identified a main effect of session in the FR_A_ [*F*(5, 340) = 14.44, *p* < 0.0001], TR_A_ [*F*(5, 345) = 31.27, *p* < 0.0001], DFR_A_ [*F*(5, 344) = 24.28, *p* < 0.0001], DTR_A_ [*F*(5, 349) = 35.69, *p* < 0.0001], and T_intr_A_ [*F*(5, 342) = 15.39, *p* < 0.0001]. There was a main effect of progression to prodromal AD (group-status) in the FR_A_ [*F*(1, 76) = 51.26, *p* < 0.0001], TR_A_ [*F*(1, 72) = 107.87, *p* < 0.0001], DFR_A_ [*F*(1, 77) = 68.38, *p* < 0.0001], DTR_A_ [*F*(1, 74) = 116.83, *p* < 0.0001], and T_intr_A_ [*F*(1, 65) = 62.50, *p* < 0.0001]. There was also a main interaction of group-status*session (Table 3) in the FR_A_ [*F*(5, 343) = 12.83, *p* < 0.0001], TR_A_ [*F*(5, 345) = 26.37, *p* < 0.0001], DFR_A_ [*F*(5, 344) = 22.78, *p* < 0.0001], DTR_A_ [*F*(5, 349) = 34.81, *p* < 0.0001], and T_intr_A_ [*F*(5, 342) = 15.69, *p* < 0.0001]. Post-hoc tests showed that the longitudinal performance of the progressors was significantly lower than that of the stable participants from session M0 to M60 on the FR_A_, from session M12 to M60 on the TR_A_ and DTR_A_, and T_intr_A_ (except M36), and from session M24 to M60 on the DFR_A_.

**Table 3:** Comparison of the 2 groups of the stable/progressors model to assess the effect of progression to prodromal AD on the longitudinal performance of the free and cued selective reminding test (FCSRT) and memory binding test (MBT) main scores.

	M_0_		M_60_			
	Stable	Progressors	Stable	Progressors		
Score name	Aβ+ (*n* = 73)	Aβ+ (*n* = 15)	Aβ+ (*n* = 52)	Aβ+ (*n* = 8)		
	Mean ± SD	Mean ± SD	Mean ± SD	Mean ± SD	*F*_df1, df2_ *=* value; *p* = value	
**FCSRT**						
FR_A_ [0–48]	30.1 ± 4.7	24.4 ± 6.3	31.0 ± 5.3	15.5 ± 5.7	**gr:** *F*_1, 76_ = 51.3; *p* < 0.001^***,a,1^ **sess:** *F*_5, 340_ = 14.4; *p* < 0.001^***^ gr*sess: *F*_5, 340_ = 12.8; *p* < 0.001^***.b,2^	
DFR_A_ [0–48]	11.8 ± 2.3	9.9 ± 2.8	11.8 ± 2.5	3.0 ± 2.7	**gr:** *F*_1, 77_ = 68.4; *p* < 0.001^***, a,1^ **sess:** *F*_5, 344_ = 24.3; *p* < 0.001^***^ gr*sess: *F*_5, 344_ = 22.8; *p* < 0.001^***,b,4^	
TR_A_ [0–48]	46.3 ± 1.7	44.7 ± 2.2	46.3 ± 1.9	34.9 ± 7.1	**gr:** *F*_1, 72_ = 107.9; *p* < 0.001^***,a,1^ **sess:** *F*_5, 345_ = 31.3; *p* < 0.001^***^ gr*sess: *F*_5, 345_ = 26.4; *p* < 0.001^***,b,3^	
DTR_A_ [0–48]	15.8 ± 0.5	15.4 ± 0.7	15.8 ± 0.5	11.6 ± 2.8	**gr:** *F*_1, 74_ = 116.8; *p* < 0.001^***,a,1^ **sess:** *F*_5, 349_ = 35.7; *p* < 0.001^***^ gr*sess: *F*_5, 349_ = 34.8; *p* < 0.001^***,b,3^	
T_intr_A_	0.9 ± 1.5	1.6 ± 2.0	0.8 ± 1.2	6.9 ± 5.6	**gr:** *F*_1, 65_ = 62.5; *p* < 0.001^***,a,1^ **sess:** *F*_5, 342_ = 15.4; *p* < 0.001^***^ gr*sess: *F*_5, 343_ = 15.7; *p* < 0.001^***,b,3^	
**MBT**						
FR_A + B_ [0–32]	17.8 ± 4.0	12.4 ± 4.2	18.7 ± 5.1	8.8 ± 5.0	**gr:** *F*_1, 80_ = 49.7; *p* < 0.001^***,a,1^ **sess:** *F*_5, 340_ = 1.6; *p* = 0.16 gr*sess: *F*_5, 340_ = 3.9; *p* = 0.002^**,2^	
DFR_A + B_ [0–32]	18.7 ± 4.9	13.0 ± 7.3	20.3 ± 5.1	8.4 ± 5.2	**gr:** *F*_1, 74_ = 54.0; p < 0.001^***, a,1^ **sess:** *F*_5, 296_ = 2.7; p = 0.02^*^ gr*sess: *F*_5, 296_ = 3.5; *p* = 0.004^***, b,2^	
TIP_A + B_ [0–32]	27.1 ± 2.4	24.3 ± 4.1	28.0 ± 3.2	19.8 ± 6.1	**gr:** *F*_1, 76_ = 52.2; *p* < 0.001^***,a,1^ **sess:** *F*_5, 293_ = 5.4; *p* < 0.001^***^ gr*sess: *F*_5, 294_ = 7.3; *p* < 0.001^***,b,2^	
DTIP_A + B_ [0–32]	27.3 ± 2.3	24.1 ± 4.1	27.9 ± 3.5	18.4 ± 5.9	**gr:** *F*_1, 76_ = 58.6; *p* < 0.001^***, a,1^ **sess:** *F*_5, 294_ = 7.5; *p* < 0.001^***^ gr*sess: *F*_5, 294_ = 7.4; *p* < 0.001^***, b,2^	
T_intr_A + B_	2.2 ± 1.9	4.1 ± 3.2	1.8 ± 1.7	9.6 ± 7.4	**gr:** *F*_1, 80_ = 33.6; *p* < 0.001^***,a,1^ **sess:** *F*_5, 340_ = 13.9; *p* < 0.001^***^ gr*sess: *F*_5, 341_ = 9.4; *p* < 0.001^***,b,2^	

*N* = neurodegeneration status; Aβ = amyloid-β status; SD = standard deviation; df = degrees of freedom; FR = free recall; DFR = delayed free recall; TR = total recall; DTR = delayed total recall; T_intr = total intrusions; TIP = total number of items recalled in the paired condition (ListA + B); DTIP = delayed TIP. The values within the brackets represent the range of possible scores per measure. The subscript A represents the FCSRT 1 list of words, and A + B represents the MBT 2 lists of words. The table shows the longitudinal mean and SD for each group in the stable/progressor model at M0 and M60. The F-values and p-values of the interaction group_status*session corresponding to the longitudinal analysis of the stable/progressor model assessed with linear mixed-effect models are represented. Significance was reported for each outcome variable based on the following *p*-values: *** *p* < 0.001; ** *p* < 0.01; * *p* < 0.05. Superscripts represent cases where significant differences were found and further assessed with emmeans post hoc analysis (corrected for multiple comparisons) as follows:

(a) cases where main effect of group was significant; progression to prodromal AD (stable > progressors):

(1) *p* < 0.001: FR_A_, TR_A_, DFR_A_, DTR_A_, T_intr_A_, FR_A + B_, TIP_A + B_, DFR_A + B_, DTIP_A + B_, and T_intr_A + B_.

(b) Cases where interactions were significant; progression to prodromal AD (stable > progressors) was observed in the following sessions:

(2) All sessions from M0: FR_A_, FR_A + B_, TIP_A + B_, DFR_A + B_, DTIP_A + B_, and T_intr_A + B_ (except M12).

(3) All sessions from session M12: TR_A_; DTR_A_; T_intr_A_ (except M36).

(4) All sessions from session M24: DFR_A_.

In the MBT, we identified a significant main effect of session on TIP_A + B_ [*F*(5, 393) = 5.41, *p* < 0.0001], DFR_A + B_ [*F*(5, 296) = 2.68, *p* = 0.02], DTIP_A + B_ [*F*(5, 294) = 7.54, *p* < 0.0001], and T_intr_A + B_ [*F*(5, 340) = 13.89, *p* < 0.0001]. There was a main effect of progression to prodromal AD (group-status) in the FR_A + B_ [*F*(1, 80) = 49.69, *p* < 0.0001], TIP_A + B_ [*F*(1, 76) = 52.22, *p* < 0.0001], DFR_A + B_ [*F*(1, 74) = 74.04, *p* < 0.0001], DTIP_A + B_ [*F*(1, 76) = 58.62, *p* < 0.0001], and T_intr_A + B_ [*F*(1, 81) = 33.57, *p* < 0.0001]. Finally, there was a significant interaction of group-status*session in FR_A + B_ [*F*(5, 340) = 3.85, *p* = 0.002], TIP_A + B_ [*F*(5, 294) = 7.34, *p* < 0.0001], DFR_A + B_ [*F*(5, 296) = 3.49, *p* = 0.004], DTIP_A + B_ [*F*(5, 294) = 7.38, *p* < 0.0001], and T_intr_A + B_ [*F*(5, 341) = 9.41, *p* < 0.0001]. *Post hoc* tests showed that the longitudinal performance of the progressors was significantly lower than that of the stable groups from M0 (until 5-years prior to diagnosis) on FR_A + B_, TIP_A + B_, DFR_A + B_, DTIP_A + B_, and T_intr_A + B_ (except M12).

### 3.4 Diagnosis accuracy of the MBT versus the FCSRT in the stable/progressors model

**Figure 4:**
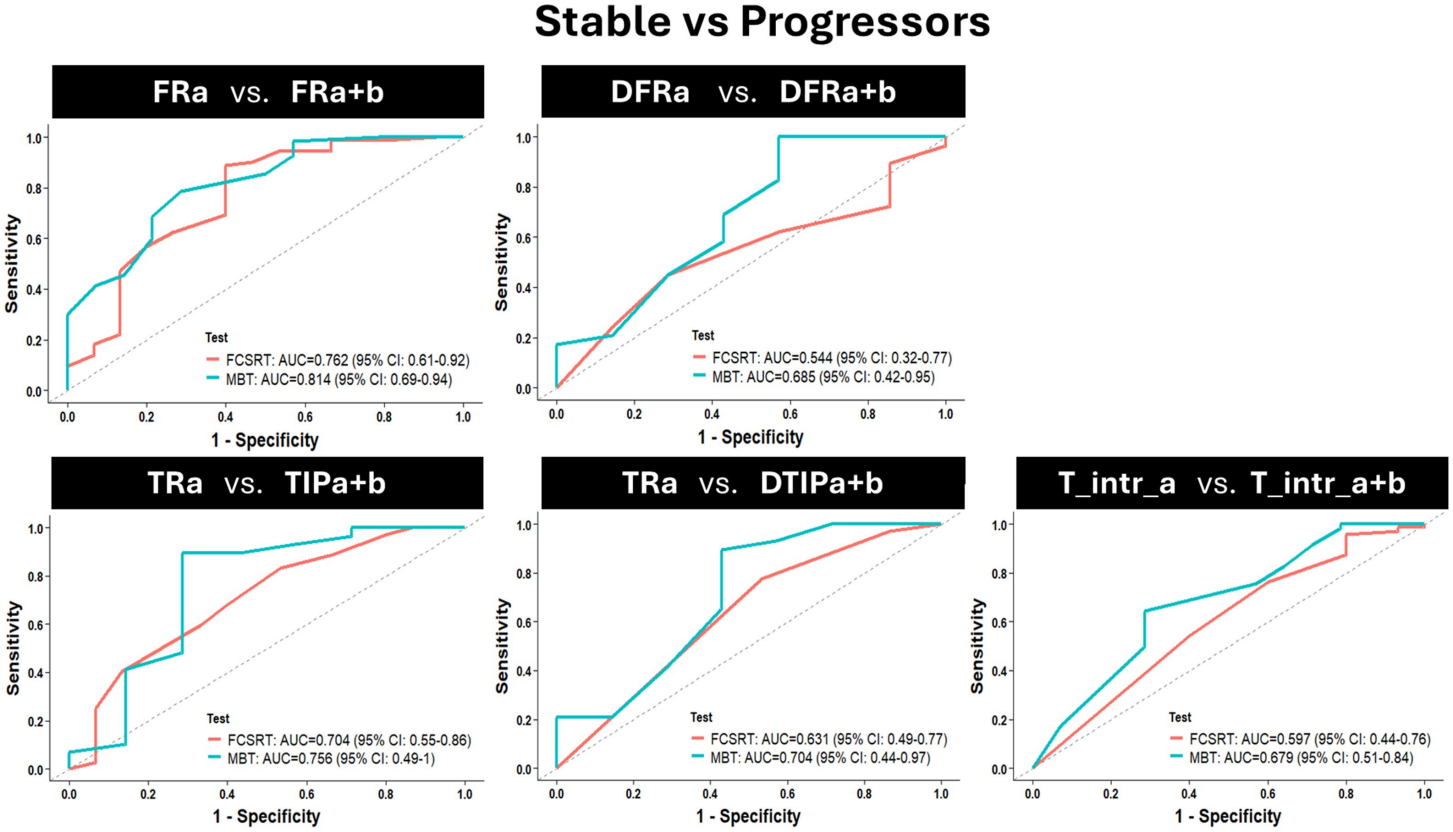
Baseline (session M0) assessment of the AD diagnosis capacity of each main score of the MBT when compared with its equivalent in the FCSRT by comparing the receiver operating characteristic (ROC) curves of each score considering the contrast stable vs. progressor groups. Each graph represents the comparison of 2 ROC curves [(1-specificity)/sensitivity], per score (a ROC curve for the score of the MBT vs. a ROC curve for the equivalent FCSRT score). Abbreviations: FR = Free Recall; TR = Total Recall; TIP = Total number of items correctly recalled in the paired condition; DFR = delayed FR; DTR = delayed TR; DTIP = delayed TIP; T_intr = Total intrusions; a = the list of the FCSRT; a + b = the two lists of the MBT.

The diagnosis accuracy distinguishing stable from progressor groups was analyzed through ROC curves for each score at baseline (Figure 4). Each graph represents the comparison between the ROC curve of one main score of the MBT with the ROC curve of its equivalent score on the FCSRT. These figures are supported by Table 4 showing comparisons at baseline for the main indices of the ROC analysis (i.e., specificity, sensitivity, accuracy, the best cut-off, and AUC) between the stable and the progressor groups. Although there were no significant differences between the tests when comparing the AUC’s of each score, there was a trend for the difference between the AUC of the DTR_A_-FCSRT and its MBT equivalent DFR_A + B_ (*p* = 0.08). In the scores associated with the AD-AHS, the MBT showed a better accuracy than the FCSRT (MBT vs. FCSRT, TIPF_A + B_/TRa = 0.89 vs. 0.50, DFR_A + B_/DFR_A_ = 0.83 vs. 0.77, DTIP_A + B +_/DTR_A_ = 0.83 vs. 0.72) and better sensitivity, --correct identification of progressors-- (MBT vs. FCSRT, TIP_A + B_/TR_A_ = 1.00 vs. 0.45, DFR_A + B_/DFR_A_ = 0.93 vs. 0.83, DTIP_A + B +_/DTR_A_ = 0.90 vs. 0.78).

**Table 4:** Baseline (sessionM0) assessment of the AD diagnosis capacity of each main score of the MBT when compared with its equivalent in the FCSRT between the stable and the progressor groups.

	Stable vs. Progressors				
FCSRT vs. MBT	Specificity	Sensitivity	Accuracy	Cut-off	AUC
FRa vs. FR_A + B_	0.60 vs. 0.71	0.89 vs. 0.79	0.84 vs. 0.77	24.5 vs. 14.5	0.76 vs. 0.81 *p* = 0.56
TRa vs. TIP_A + B_	0.71 vs. 0.43	0.45 vs. 1.00	0.50 vs. 0.89	46.5 vs. 22.5	0.54 vs. 0.69 *p* = 0.30
DFRa vs. DFR_A + B_	0.47 vs. 0.71	0.83 vs. 0.93	0.77 vs. 0.86	9.5 vs. 13.5	0.70 vs. 0.76 *p* = 0.085
DTRa vs. DTIP_A + B_	0.47 vs. 0.57	0.78 vs. 0.90	0.72 vs. 0.83	15.5 vs. 24.5	0.63 vs. 0.70 *p* = 0.12
T_intr_ vs. T_intra__A + B_	0.40 vs. 0.71	0.76 vs. 0.64	0.70 vs.0.65	1.5 vs. 2.5	0.59 vs. 0.68 *p* = 0.31

The table represents the comparison of the main indices of the ROC analysis between the two tests per score. AUC = Area Under the Curve; FR = Free Recall; TR = Total Recall; TIP = Total number of items correctly recalled in the paired condition; DFR = delayed FR; DTR = delayed TR; DTIP = delayed TIP T_intru = Total intrusions. The subscript A represents the FCSRT 1 list of words, and A + B represents the MBT 2 lists of words. Significance was reported for each outcome variable based on the following *p*-values: *** *p* < 0.001. ** *p* < 0.01. * *p* < 0.05.

## 4 Discussion

Overall, our results reiterate our previous preliminary work confirming the capacity of the MBT to anticipate the detection of AD-linked EM deficits to an asymptomatic at-risk stage, while EM capacity remains normal according to FCSRT cut-offs (official AD-diagnosis tool; Raposo Pereira et al., 2024). This was reflected in the higher number of scores detecting EM deficits linked to AD ± 4 years prior to a prodromal diagnosis. Moreover, while neurodegeneration in isolation did not seem to influence EM performance, Aβ burden in isolation and its cumulative effect with neurodegeneration seemed to exert opposite effects in the total number of MBT intrusions.

From observation, in the first stage of analysis both the FCSRT and MBT followed a similar performance pattern in controls, according to expectations (Auriacombe et al., 2010; Gramunt et al., 2015, 2016; Papp et al., 2015; Grober et al., 2018; Mowrey et al., 2018). In the FCSRT, the FR score was lower than TR score, and FR and TR scores were higher than their delayed versions (Auriacombe et al., 2010; Papp et al., 2015, 2017; Grober et al., 2018). In the MBT, the FR score was also lower than the TIP score, but no differences were found between their immediate and delayed versions (Gramunt et al., 2015, 2016; Mowrey et al., 2016, 2018; Buschke et al., 2017; Gagliardi et al., 2019). These findings suggest that both tests met the appropriate level of difficulty for the cognitive capacity in this population.

When testing Aβ burden and neurodegeneration, we found an expected cumulative effect of these biomarkers, where the participants with both biomarkers showed more intrusions on the MBT than the stable/N- group. This contrasted with an effect of Aβ burden alone in the direction contrary to the expected, with controls (Aβ-) showing more MBT intrusions than the stable/N- (Aβ+) group. A deleterious cumulative effect of Aβ burden and neurodegeneration is in line with previous work from our group and others, which has been associated with the onset and exacerbation of EM decline in AD, but has not been extensively explored in association to binding scores (Papp et al., 2015, 2017; Bilgel et al., 2018; Chipi et al., 2019; Gagliardi et al., 2019). One possible explanation for this cumulative effect might be that neurodegeneration (as an expression of decreased glucose uptake), appearing later in the AD continuum after the appearance of Aβ burden, increases neuronal susceptibility to Aβ-linked toxicity, thereby reducing the cognitive resources available for EM processing (Sperling et al., 2011; Papp et al., 2015, 2017; Bilgel et al., 2018; Chipi et al., 2019). In contrast, a higher number of intrusions from the controls versus the stable/N- group was unattended and might perhaps be an expression of a compensatory mechanism linked to the particularly high average level of education of this sample (Rentz et al., 2013; Arenaza-Urquijo et al., 2017; Papp et al., 2017; Bilgel et al., 2018; Stern et al., 2020; Cecchini et al., 2021). High education has been suggested to prompt a cognitive reserve process capable of sustaining normal cognition in individuals with Aβ burden for longer (Arenaza-Urquijo et al., 2017; Bilgel et al., 2018; Stern et al., 2020). Therefore, it is a possibility that in the stable/N- participants with a certain amount of Aβ pathology but without neurodegeneration, their cognitive reserve could have been potentially delaying symptomatology before neural decline becomes overwhelming and starts uncovering EM impairment (Arenaza-Urquijo et al., 2017; Bilgel et al., 2018; Stern et al., 2020). However, the scope of this study did not allow us to confirm this hypothesis.

The influence of progression to prodromal AD on FCSRT and MBT performance was notable. As expected, FCSRT deficits were initially observed in the FR, appearing only 1 year later in the TR and their delayed versions (Auriacombe et al., 2010; Gramunt et al., 2016; Grober et al., 2018; Raposo Pereira et al., 2024). This underlies one of the major problems with the FCSRT in anticipating AD diagnosis in the earlier preclinical stages (Auriacombe et al., 2010; Gramunt et al., 2016; Papp et al., 2017; Grober et al., 2018; Loewenstein et al., 2018; Raposo Pereira et al., 2024). The fact that FR in isolation, an ‘accessibility’ measure mostly linked to executive or attention deficits, is not necessarily AD-specific (Auriacombe et al., 2010; Gramunt et al., 2016; Papp et al., 2017; Loewenstein et al., 2018). Therefore, only when decline is observed in TR, a measure of ‘availability’ underlying the AD clinical phenotype (AHS), but also in memory consolidation measures (i.e., DFR and DTR), can EM deficits be specific to AD (Dubois and Albert, 2004; Auriacombe et al., 2010; Sarazin et al., 2010; Gramunt et al., 2016; Papp et al., 2017; Loewenstein et al., 2018; Raposo Pereira et al., 2024). Furthermore, TR decline tends to appear later, only after FR deficits, largely because of the ceiling levels associated with the use of only one list of words (Grober et al., 2000; Auriacombe et al., 2010; Papp et al., 2017; Loewenstein et al., 2018; Raposo Pereira et al., 2024). This restricts the comparison of TR scores with cognitive aging equivalents, preventing the detection of variations in maximum recall until deficits are too advanced and semantic cues can no longer overcome them (Auriacombe et al., 2010; Sarazin et al., 2010; Gramunt et al., 2016; Papp et al., 2017; Loewenstein et al., 2018; Raposo Pereira et al., 2024). Such was not the case in the MBT, where variation in maximum recall was observed up to 4 years prior to AD-diagnosis, and EM deficits were observed not only in the FR, but in all the AD neural correlated scores linked to the paired condition and rooted in the hippocampus (i.e., binding and consolidation measures; Gramunt et al., 2016; Papp et al., 2017). Moreover, despite the model performance (AUC) for the MBT scores when compared to the equivalent scores of the FCSRT was always better, there were no significant differences in performance between the tests for any of the scores. However, when assessing qualitatively the trade-off between specificity (correct clearance of AD-diagnosis) and sensitivity (correct AD-diagnosis), the sensitivity (as well as the accuracy) of the binding and consolidation scores, suggested as more distinctive of AD-linked EM deficits, was always remarkably higher (Gramunt et al., 2015; Buschke et al., 2017; Papp et al., 2017; Mowrey et al., 2018). Therefore, our results suggest that the MBT might be more sensitive and more accurate than the FCSRT, by correctly identifying the progressors already at baseline, though these results should be interpreted carefully due to the small sample size of the progressors group.

Evidence from our group and others has suggested that the sensitivity of the MBT to anticipate diagnosis at an asymptomatic at-risk stage while individuals are still cognitively normal is higher when compared with other EM tests, including the FCSRT, one of the most widely used official diagnostic EM tools (Gramunt et al., 2015; Buschke et al., 2017; Papp et al., 2017). Here, we confirmed this hypothesis by showing the capacity of the MBT versus the FCSRT to anticipate diagnosis based on a higher number of AD neural correlated scores.

This longitudinal, multimodal, and monocentric study presented a great opportunity to retrospectively assess in a considerably large sample of at-risk for AD individuals the development of EM deficits and the progression to AD (Dubois et al., 2018). The use of the MBT employing two lists of words allowed the assessment of associative memory, more precisely ‘binding’, a component that has been suggested as more sensitive to the emergence of subtle EM alterations linked to AD, but nevertheless remains largely understudied. Furthermore, the use of two EM tests, employing similar cue-learning and recall procedures, resulted in the advantage of having FR and TCR scores that can be compared between the tests. Overall, the results suggest that in a sample with different levels of AD biomarkers the MBT seems to present a higher accuracy (and sensitivity) when compared with the FCSRT, in the identification of the individuals that will become prodromal in at least 4 years prior to diagnosis.

Despite its strengths, this study has some important limitations and the findings presented here should be interpreted with some caution. One main limitation is the small sample size of the progressors group. Although the valuable knowledge obtained from the opportunity to follow-up the at-risk stage of these participants for 5 years, these findings need to be replicated in larger samples in order to reinforce our conclusions. Furthermore, the comparison of FCSRT and MBT performance was restricted to the comparison of their main scores and the onset session where deficits started to occur. Admittedly, a more detailed comparative analysis could offer a more precise picture of their performance differences. Although equivalent, the parallel convergence between the scores of the two tests should be interpreted carefully. Another potential disadvantage was the high average level of education in the cohort, which does not represent the normal aging population. In addition, although we controlled for the potential effects of age, education, and sex, their residual influence may still be present.

Extending the previous preliminary work initiated by our group, this study builds on evidence suggesting the advantage to use the MBT as an official diagnostic tool that can anticipate the detection of AD neural correlates of EM deficits to an asymptomatic at-risk stage (Raposo Pereira et al., 2024). In a period where individuals remain cognitively normal according to FCSRT normative cut-offs and present with no conscious perception of any objective cognitive or executive decline by either themselves or their companions (Auriacombe et al., 2010; Gramunt et al., 2015; Papp et al., 2017; Bilgel et al., 2018). The MBT seems capable of more accurately predict who will progress to AD, increasing the number of years prior to diagnosis in which EM deficits are detected and the number of scores linked to the AHS-AD clinical phenotype in which decline is detected. This shows the potential of the MBT as an initial screening test and offers a unique opportunity to apply promising therapeutics at an early preclinical stage, when cognitive decline has a higher potential to be successfully delayed and the quality of life prolonged.

## Data availability statement

The original contributions presented in the study are included in the article/Supplementary material, further inquiries can be directed to the corresponding author.

## Ethics statement

The studies involving humans were approved by guidelines of the Helsinki Declaration Ethical Principles for Medical Research involving Human Subjects, Tokyo 2004; the ethics committee of the Pitie-Salpêtrière University Hospital; INSIGHT-PreAD protocol and the INSIGHT-preAD scientific committee of the Pitie-Salpêtrière University Hospital. The studies were conducted in accordance with the local legislation and institutional requirements. The participants provided their written informed consent to participate in this study.

## Author contributions

FR: Conceptualization, Data curation, Formal analysis, Investigation, Methodology, Project administration, Software, Validation, Visualization, Writing – original draft, Writing – review & editing. NG: Funding acquisition, Resources, Supervision, Validation, Writing – review & editing. GD: Conceptualization, Writing – review & editing. BD: Conceptualization, Funding acquisition, Validation, Writing – review & editing. VL: Conceptualization, Data curation, Formal analysis, Funding acquisition, Methodology, Project administration, Resources, Supervision, Validation, Visualization, Writing – review & editing.
