# Supplementary material for: The memory binding test can anticipate Alzheimer’s disease diagnosis at an early preclinical stage: a longitudinal study in the INSIGHTpreAD cohort
Source: Front Aging Neurosci. 2024 Aug 8;16:1414419. doi: 10.3389/fnagi.2024.1414419 (PMC11340525; doi:10.3389/fnagi.2024.1414419)

**Supplementary material**

**Figure_1S Individual response to each of the main measures of the Free and Cued Selective Reminding Test (FCSRT) and of the Memory Binding Test (MBT) per session divided by groups according with the Aβ burden/Neurodegeneration model**. Each graph shows the violin plot representing the individual responses to the main scores of the FCSRT and the MBT distributed per session and per group (i.e. controls = green, stable/N- = blue, stable/N+ = purple). Each line represents the average response per group per session.


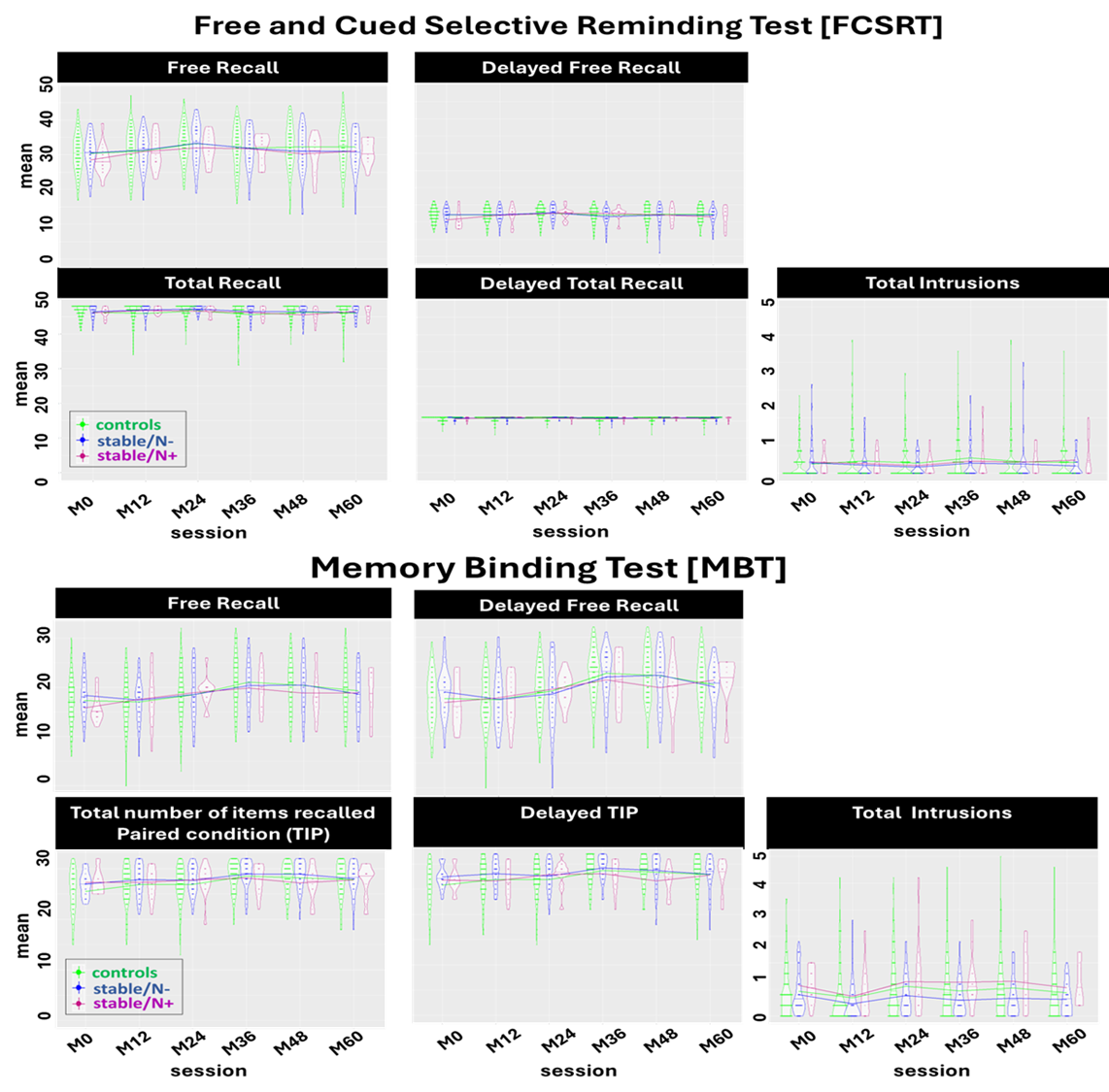


**Figure_2S Individual response to each of the main measures of the Free and Cued Selective Reminding Test (FCSRT) and of the Memory Binding Test (MBT) per session divided by groups according with the Stable/Progressors model.** Each graph shows the violin plot representing the individual response to the main scores of the FCSRT and the MBT distributed per session and per group (i.e. stable = blue, progressors = red). Each line represents the average response per group per session.


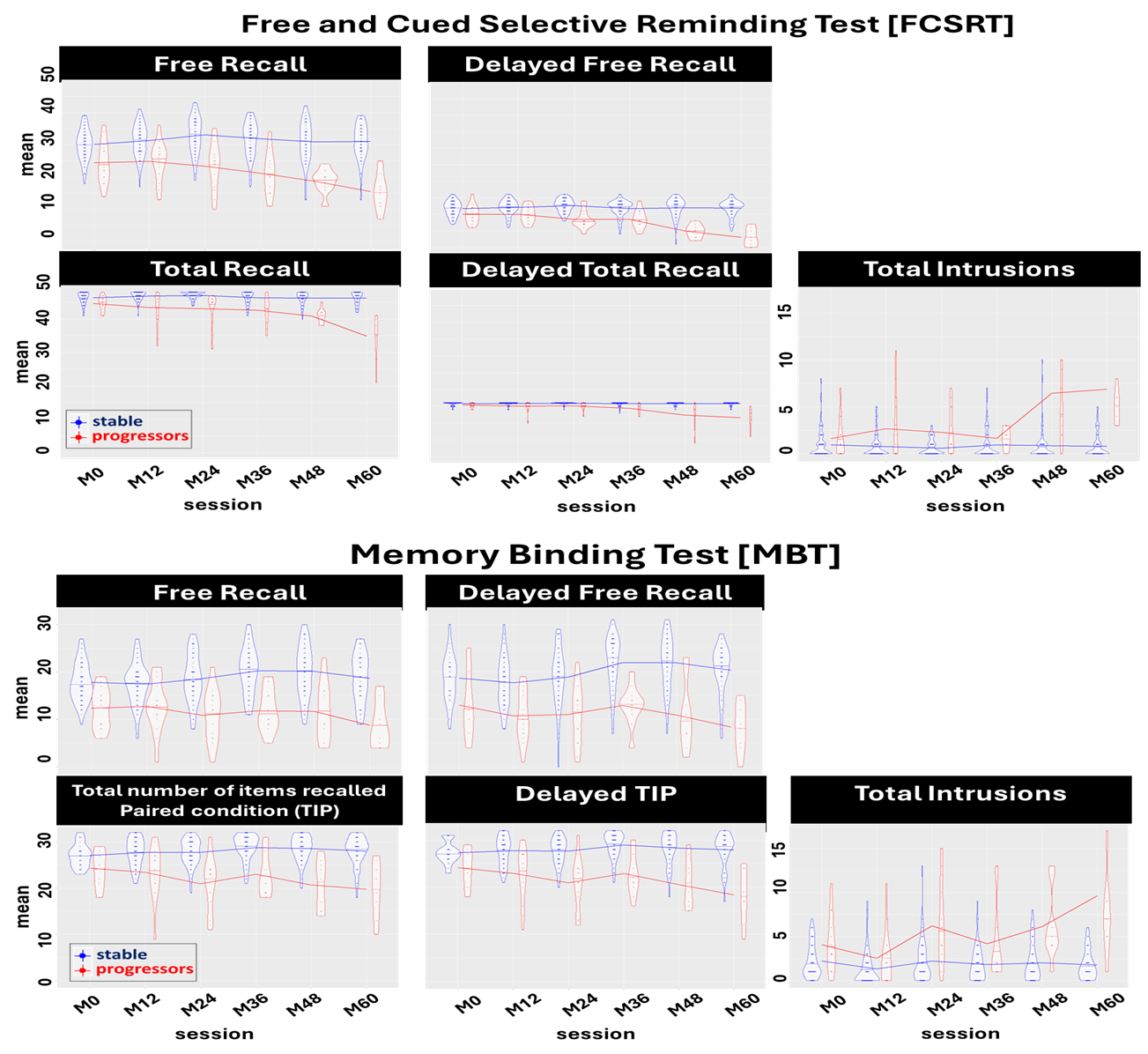

Supplement: Supplementary file 1 [file Table_1.DOCX]
